# United States College of Veterinary Surgeons

**Published:** 1897-05

**Authors:** 


### UNITED STATES COLLEGE OF VETERINARY SURGEONS.

The annual examinations of this institution, which have been in progress since April 1st, closed on the 15th. The Faculty presented the following gentlemen for graduation, they having completed the course and passed successfully the prescribed examinations in all branches of veterinary science: Webster Clay Langdon, of Fargo, N. D.; William B. Elliott, V.S., of Riverside, Cal.; William E. Yetton, of Washington, D. C.

The Hon. J. H. Brigham, Assistant Secretary of Agriculture and Master of the National Grange of the United States, was introduced to the Faculty and students by the Dean, Prof. C. Barnwell Robinson, after having opened the ceremonies of commencement with a brief but interesting outline of the history and progress of the College.

Secretary Brigham presented the diplomas to the graduates with a few very appropriate words in which he pointed out the close relation between the veterinarian and the agriculturist, and emphasized the necessity of profiting by their education to make their experience of use to them, of a high standard of rectitude in their relations with their clients, and of humanity toward their patients, thus being true to themselves and to their alma mater.

Prof. D. S. Lamb, A.M., M.D., then delivered on behalf of the Faculty the following address:

At the request of our worthy Dean, I will say a few parting words in this, the closing hour of the third session of the college and the corresponding commencement.

Since a year ago, during which teachers and pupils have been devoting themselves to the routine of lectures and recitations, clinics and laboratory work, the world of medicine, both in its human and comparative side, has been making progress. This progress has been most marked in the domains of pathology, diagnosis, and treatment. Pathology has been notably enriched through the work of the experimental laboratory, and especially by the investigations in bacteriology. The study of the pathogenic organisms; their multiplication, especially in the form of spores; the methods, especially the methods of staining, by which they may individually be recognized; their different forms at different periods and under different circumstances, especially their involution-forms; their strong resemblances and marked differences, and their cultures, by which also they may be identified; their habits and habitats; their relative and actual virulence; the various ways by which they cause death; how they may be destroyed, from the air, from the water, from the soil, from our clothing, houses, and food; from the animal itself; how they may be attenuated or intensified; the relative and actual susceptibility of individuals to their influence and the influence of their toxic products; how long they can preserve their pathogenic powers, and under what circumstances; what is the best and most efficient prophylaxis against them, and what the best and most efficient treatment of the diseases which they cause.

These and other questions which I have not mentioned, are questions which arise and are to be answered in connection with each pathogenic organism. The number and variety of these questions will give sure ideas of the vastness of the subject and the immensity of the work which the bacteriological laboratory is called upon to do. The year's work has been large and various. Just how much of the result promises to be useful in the prevention and treatment of disease remains to be seen. Of course, in our impatient way, we are prone to forget that the progress of the world is in the main by slow and laborious steps. It is only at intervals that we are dazzled by some great and brilliant discovery, as by the comet in the depths of the space around us. So also in our lives, when we sum up the year's work in a pecuniary way, after deducting our expenditures from our receipts, our actual savings may be very small indeed. So also in science, the sum total of progress in the year may seem very small. But the work has been done, it has been good work and useful, and at the end of the year there is a gain, though it may seem disproportionately small.

Perhaps the most interesting and important part of this subject of bacteriological work is that of serums, their prophylactic and curative powers. The subject is broad and deep, the results thus far obtained are at the least very encouraging. We have learned one great truth beyond any question, and that is that a healthy blood-serum is very decidedly germicidal, so much so that the bacteriologist often fails to grow his organism upon it. Thus with the addition of leucocytes, we are, so to speak, twice armed against the germs that may be introduced within our systems. By means of certain methods and processes we are able to procure serums which when introduced into the animal economy are found to prevent or to act as an antidote to certain diseases. Especially among the lower animals has this new truth a great commercial value, apart from what I may call its humanity. How these animals may be inoculated; how they may be immunized; how their early infection with disease may be diagnosticated; how they may be cured by means of attenuated toxins and antitoxins. These are some of the questions and problems which confront us and which are being answered one by one for now this disease and then that.

I am aware that this subject of microöganisms is perhaps becoming tiresome, especially to those who are yet skeptical in regard to it, but it seems to me that the revelations of the microscope and of the staining-methods and cultures and experiments of the bacteriological laboratory are not fairly appreciated even by the most enthusiastic students. The light is as yet too dim to see much of this, as well as of other vast vistas in science. I believe that we as yet but remotely conceive the far-reaching expansion of our vision in the next decade.

I pass by the other items of the pathological advances of the year, and will touch for a moment upon the work of the clinical laboratory. This indeed has revolutionized and is revolutionizing the subject of diagnosis, especially in human medicine. This subject, too, is too vast to dwell upon long. The study of the blood-cells, the leucocytes, the red cells, the plaques, etc., is helping us to a new and unexpected means of diagnosis, which in its way promises as much variety as the other subjects of bacteriology—the number of cells and of each kind of cell in the cubic millimetre of blood at various times, as after a night's rest, after a meal, after meals of certain definitely known constituents, under circumstances of active exercise, of sleep, of sedentary work, in the thousand and one diseases to which man and the lower animals are liable. Not only the number of these cells, varying on occasion, but their size, and shape, mononucleated, multinucleated; their response to various staining-reagents—here is an almost infinite variety. We are only on the threshold of the great building which our children and our children's children are destined to see. There is now added to our means of diagnosis an increasingly powerful and certain help, the value of which is too dimly conceived. I could dwell upon this topic a long time, but dare not.

The diagnosis of diseases of the stomach, in the human subject especially, is now greatly aided by this same clinical laboratory, by the giving of test-meals and their subsequent examination, to determine the digestive power of the stomach; the examination of the stomach-contents in important cases, in medico-legal as well as individually, simply diagnostic. Thus, in regard to the diagnosis of diseases of the kidney, we have the conjoined examination of the urine and the blood. A little blood from the end of the finger may settle the question of malaria, or typhoid fever, or some other disease. A little blood and a little urine may determine whether we have a malignant disease of the urinary passages; permits us to estimate the probabilities of a generalization of the malignant growth.

Again, in the clinical laboratory in conjunction with the physiological, we may determine the action of medicine and diseases under the most favorable circumstances, so far as the lower animals are concerned, and by analogy we may reason out more or less accurate conclusions for the human subject; the results are promising. Taken altogether, the laboratory work of to-day is carrying the modern physician to a place in practical diagnosis and therapeutics beyond the brightest dreams of the fathers in medicine. To this may be added the progress in sanitation, the municipal, State, and personal means by which we may protect our homes; schools and other buildings may be improved in matters of light, of heat, of ventilation; our water-supply purified; our food-supply preserved unadulterated, and our medicines as well; and the thousand other ways by which our municipal and individual health may be preserved, our life lengthened.

One by one an enemy falls, and the time should come, and probably will, when the last one shall perish by the wayside.

New industries, new inventions, introduce new dangers which physician and sanitarian must prepare to meet. We have each an individual duty to perform, the proper diffusion of scientific knowledge is helping toward the good time coming, because as the laity are educated they co-operate with the physician and sanitarian.

Evidently there is a very definite progress each year in the light of municipal aad personal rules of health. Our homes are so much better lighted, heated, ventilated, than those of our fathers; our dietaries improved, our clothing more healthful, provided that fashion will permit.

We bathe a dozen times where our fathers bathed once. We have improved at the same time the housing and surrounding of our domestic animals. Cleanliness, which is so close to godliness, is a thousand times better observed in our case than in older times. A word to the gentlemen who graduate.

I can enter somewhat into your feelings of the moment, as you think of the transition you are making from the state of pupilage to that of the practitioner. You have a future, and you hope it may be a bright and happy one; and I hope so, too. But my own experience and observation have shown me that there are two things you ought to make up your minds to do. One is to lead a straightforward, honest, and manly life, despising mean and little things, and trying to do the best you can—good practitioners and good citizens, trying to improve your time and opportunity, commanding the respect and confidence of your fellows. The other is to remember, as the lamented Garfield was wont to say, "It is the unexpected that happens," or as Cardinal Wolsey said:

> . . . comes a frost, a killing frost,
> And when he thinks, good easy man, full surely
> His greatness is a-ripening, nips his root,
> And then he falls.

Or that other quotation, the author of which I cannot at this moment recall, and whom you will probably recognize as familiar to you:

> "From care and trouble rest your thought,
> Even when the end's attained,
> For all your plans may come to naught
> When every nerve is strained."

These things are not said to discourage you. Life's successes are attained by honesty, by diligence, good judgment, industry accompanied by a certain indispensable amount of knowledge of your profession, some more, some less. If you preserve your health, and I hope you will, keep yourselves free from bad habits and observe the other things I have mentioned—you will succeed, and you will deserve to succeed.

I express the sentiment of the entire Faculty, whom I to some extent represent, in commending you to the kindness and moral support of your fellow practitioners, while you attend diligently to the duties of your vocation.

The Valedictorian of the class, Mr. Langdon, then addressed the Faculty and the students thus:

HONORABLE THE DEAN, MEMBERS OF THE FACULTY, AND FELLOW-STUDENTS.

GENTLEMEN: I thank you for the privilege and honor extended to me in giving me the opportunity to act as spokesman in delivering the farewell address to the Faculty for the class of '97.

I regret that the exigencies of the case have prevented me from preparing a more elaborate address for the occasion for extemporaneous delivery, but the preparation for the final examinations in a college whose curriculum has undoubtedly the widest range of any similar school in this country, embracing as it does fifteen subjects upon which the student must pass before receiving his degree, leaves, I assure you, but little time, and very much less energy in the individual of ordinary abilities, to commit an address to memory.

It has seemed to me that when a few sincere words of justified appreciation, where such appreciation is due, and the assurance of mutual good-fellowship, and a desire for continued advancement along the lines marked out for us by an honored and faithful Faculty, are all that could be required of me at this time.

We are as students particularly favored by the cosmopolitan character of this city, the home of our alma mater. This characteristic is well reflected in the composition of our classes, which literally include men from New Hampshire to California. Such a condition is perfectly natural, growing as it does out of the numerous and varied educational advantages afforded by the museums and other public institutions, as well as by the residence in the city of those thoroughly skilled in the various branches of learning and science here represented. Boston for years was considered the hotbed of education, in fact was conceded to hold the palm as the principal seat of learning in America. New York has been her rival for years, but Washington, with the characteristic precocity of all things American, seems to have wrested this honor from all competitors at one grasp. It is, then, our duty, and will be our pleasure in so far as this institution forms a part of a great and unique whole, to maintain this eminence, and add if possible new laurels to her fame.

And what, indeed, will be our excuse if we fail utterly in this, our obvious duty? The indomitable will, energy, discernment, and faith of our Dean have secured for us, if you will pardon the directness and flatness of the assertion, the flower of the profession in each particular branch included in our course—the elder men of worldwide reputation, and the younger proportionately known and appreciated, and as all our class believes, particularly gifted with the talent most useful to the world, and consequently most desirable—the ability to impart knowledge.

The institution, though young in years, is, aside from the great advantage of a competent corps of professors, equipped with the necessary appliances for practising the precepts poured out to us by our instructors, and thus if we do not leave our alma mater well supplied for our journey, it is not through any lack of depth in the well or any lack of size or force in the stream, but, on the other hand, through the shallowness of the receptacle into which it flows, and if the same honor is not in the time to come attached to the degree of this, as to that of any other school in the land, we have no one to blame but ourselves. *Ad astra per aspera*, literally "to the stars through asperity," or in the very free translation, or rather paraphrase, of Emerson, "Hitch your wagon to a star."

Honored preceptors and instructors, we bid you farewell, begging you to accept our hand of friendship and appreciation, with the assurance that your labors in our behalf shall not be in vain and shall not go unrewarded, and that it will be our aim to do our part in the upbuilding of the institution, to the end that not the least of your causes for self-congratulation shall be that you are professors thereof.

Fellow-students, we have passed these months together in mutual efforts toward the acquisition of knowledge and with mutual pleasure and good feeling. But we must not forget that our responsibilities only begin here. We are given the material for the foundation of an honorable position in our chosen calling, and in which there are ample opportunities of raising the standard of excellence, and of raising our standing in the world at large. There is no reason why we should not be on a level with all other learned professions, for to have success here are required as much learning, as much skill, and infinitely more observation and intuition than in human medicine, together with a knowledge of comparative medicine and surgery not absolutely necessary to the latter.

What are our means so far equipped of attaining this end? Few and simple indeed and plainly within our grasp—strict honor, absolute truth, "though not necessary to tell all we know about a thing at all times," conscientious work, which means the establishment of perfect confidence between practitioner and client.

Our course of prescribed study finished, we will not weakly and abjectly sink into a life of pure routine which precludes all advancement, but struggle not only to hold our footing, but try to make further progress; for, says an eminent philosopher of our times, "He who is silent is forgotten; he who abstains is taken at his word. He who does not advance falls back. He who stops is overwhelmed, distanced, crushed. He who ceases to grow greater, becomes smaller. He who leaves off, gives up. The stationary condition is the beginning of the end, is the dreadful symptom that precedes death." Therefore, let us on the eve of this new era in our lives, while looking into the future, full of dangers and difficulties, but with the shining goal in view, bear in our hearts the admonition so appropriately selected as the motto of our alma mater, "*Vestigia Nulla Retrorsum.*"

Prof. George A. Prevost then, on behalf of the trustees, made a few exceedingly felicitous remarks which were received with applause.

The second-year examinations were passed by Messrs. M. S. Lantz, Samuel Gelson, George N. Brady, Benjamin Gheen.

Mr. Arthur Currier took the prize for the best general examination in the first-year class.

The degree of Fellowship (F.U.S.C.V.S.) was conferred upon D. S. Lamb, A.M., M.D., of Washington, D. C., and C. H. Ford, D.V.S., of New Orleans, La.

The conditions under which this degree is granted may be more fully understood by reference to the catalogue and prospectus of the College.

In this instance it was conferred owing to scientific contributions from the candidates.

